# Notes of Three Cases of Aneurism, Met with in the Black Women’s Medical Ward, Philadelphia Hospital, Blockley

**Published:** 1844-04-20

**Authors:** Geo. N. Burwell

**Affiliations:** Resident Physician


### THE MEDICAL EXAMINER,

### And Record of Medical Science.

Vol. VII.] PHILADELPHIA, SATURDAY, APRIL 20, 1844. [No. 8

### NOTES OF THREE CASES OF ANEURISM,

*Met with in the Black Women's Medical Ward, Philadelphia Hospital, Blockley.*

By Geo. N. Burwell, M. D., Resident Physician.

Service of Prof. Dunglison.

[These cases occurred in the wards during the clinical session of the present winter, and were made the subject of comment by Professor Dunglison. Some of his remarks have been given in the reports of the clinic at the Philadelphia Hospital.]

Case I.—Sarah Green, aged fifty-six years, entered the ward, November 21, 1843. She had then some fever, a quick, rapid pulse, cough, bronchitic expectoration and pain, sometimes in the chest, and again in one hypochondrium, which would shoot across into the other. There was no pain or tenderness of abdomen developed on pressure. Her bowels had been regular. She laboured under great depression of spirits, and thought she would die. She had been a hard-working woman; habits temperate. The usual remedies for sub-acute bronchitis were prescrib.d.

The second time I saw her was the evening before her death, and she had then evident sub-acute peritonitis; there was no severe fever, full or hard pulse; the pain on pressure was well marked but moderate in degree, and not such as to prevent her from changing her position without much suffering. Leeches and fomentations were directed to the abdomen,

The next morning (Dec. 1) she was apparently better, and so expressed herself; she had rested pretty well during the night; her bowels had been open two or three times.

While on the close-stool at noon, she suddenly fell over, was immediately laid on her bed, and died in ten minutes without uttering a word.

*Post-mortem.*—The first points that attracted our attention on opening the cavity of the abdomen, were recent coagula of blood among the convolutions of the intestines, and especially in the right hypochondriac region, where there lay so much coagulum as to suggest the idea of the rupture of an aneurism of the upper portion of the abdominal aorta: a large quantity was also found in the cavity of the pelvis. The serum of the blood lay principally in the lumbar regions. There was marked and general injection of the peritoneal coat of the intestines. No effusion of lymph was noticed.

On cleaning out the coagula, turning out the intestines, and searching for the source of the blood, it was found to have proceeded from an aneurism of the internal iliac artery of the left side, at its very origin. The aneurism was globular, and about an inch and a half in diameter; it was filled with the usual laminæ of coagula, and infringed somewhat on the origin of the external iliac artery of the same side. It had given away on its upper surface. The cellular tissue connecting the various tissues and vessels of the part, and that over the promontory of the sacrum, was much thickened and indurated; there was also an effusion of lymph, of old standing, down the peritoneum, lining the rectum as far as the fundus uteri, which was tinged red by the blood.

Case 2.—Jane Knight, aged thirty-four years, of short stature, and generally a hard working woman, intemperate, entered the ward, December 28, 1843. The following were her prominent symptoms. The first and most obvious on looking at her, was a difficulty of breathing. This varied considerably on different days, without much apparent regularity, except in the morning about three o'clock, when, for ten or twelve days in succession before her death, she had at this hour excessive difficulty in getting her breath, and several times believed that her end had come. After thus suffering for some time, however, she would begin to expectorate, at the same time perspiring freely and in the sitting posture, and the paroxysm would pass off, leaving her much prostrated.

She had a harassing cough, and an expectoration which was peculiar. For the first week it was transparent, thick and pearly, and almost without froth. This amounted some days to a pint; at other times when she felt relieved, to not more than five or six ounces. It became more frothy later, and during the last two days, rusty.

The resonance of the chest was generally clear. Auscultation revealed the rhonchi often heard in complication of bronchitis with emphysema, the *cantus avium omnium* of Laennec, and a single cough would often change the character of all of them. These rhonchi were some days much less in amount, but never in extent, for they could be heard in every portion of the lungs; and again, without any assignable cause, they would become much aggravated.

There was more than ordinary dulness in the region of the heart, and a decidedly roughened first sound. The heart was irregular and spasmodic in its action; the impulse correspondingly quick and somewhat forcible. She had at first a slight increase of the heat of the skin, but not at all of that character and continuance belonging to frank inflammation: her pulse was that of irritation, generally hurried and feeble. She at no time complained of marked pain; the most was a sense of uneasiness or constriction about the chest. Her previous history threw no more light upon her disease than that she had had an attack similar to the present one, a few weeks before, which was relieved principally by blisters. She had not been well since, suffering most of the time with shortness of breath.

The treatment consisted of various expectorants and anodynes, but without any good effect: blisters were the only remedies which gave her relief, and these only while they were discharging. Three were applied.

Pneumonia succeeded, January 15, in the lower lobe of the left lung, and in two days thereafter she died.

The post-mortem examination revealed an aneurismal tumour of the arch of the aorta about two inches in diameter, which was filled with laminated coagula. It had not ruptured, although in one place the sac was much thinner. The coats of the aorta about its origin were much thickened, and lymph had been deposited so as entirely to obstruct the orifice of the left carotid artery. From the valves to the arteria-innominata the internal membrane of the aorta had the bright red injection of inflammation; the valves and the endocardium were unaffected by this. The aneurism was closely adherent to the œsophagus on the left, and made considerable pressure upon, and was also partially attached to, the trachea. One of the mitral valves was so much atrophied that it had but half the width of the other; there was decided hypertrophy of the left ventricle. The lower lobe of the left lung was shown to be the seat of pneumonia in the second stage.

Case 3.—Caroline Welsh, a cook, aged forty years, born in the West Indies, but has lived in this country since she was seven years of age.

Entered the ward, January 26, 1844, with a sharp pleuritic pain in the left axilla; heat of skin moderate; pulse accelerated but feeble; cough; expectoration rather abundant, of a frothy mucus, somewhat viscid, and at times tinged with blood; has kept her bed for two weeks only; generally enjoys good health, and for the last year suffered only from slight cold; is not emaciated.

Has had the cough for three months, but not as bad as at present. When the present attack commenced she "puked" blood (not coughed it up, she says) for two or three mornings, and at evening spat up blood; has never had chills, or night sweats, and the fever is of recent date.

Has been short-breathed for six years, especially when working hard; this has been *bad* for the last three and a half months; has had more or less of palpitation of the heart for the last six months, but not at all violent; has had, for some time, a pain at the junction of the second rib of the right side with the sternum, and this place is quite tender on pressure.

*Physical Signs.*—Percussion anteriorly on left side resonant over the usual space, and does not show the heart to be notably enlarged. On the right side anteriorly, the percussion flat throughout; posteriorly, more resonant on the right side over the lower lobe, than over the lower lobe left side.

*On auscultation* we find the air passes into the upper lobe of the *left lung*, while throughout the lower lobe and axilla (in the seat of the pain) a rather fine submucous rhonchus is heard.

*Right Lung.*—Over the upper lobe, *anteriorly*, can hear scarcely anything except on making the patient distend her chest well and rapidly, when a very *feeble* sound is heard, approaching to bronchial respiration. The resonance of the voice here is also an approach to bronchophony, but not near as distinct as we hear in tubercular consolidation of the lung of this part, where the percussion is so perfectly flat as it is here. *Posteriorly*, the vesicular murmur is heard.

On ascultating the heart, found the *first* sound roughened; the *second* distinct; the impulsion not at all exaggerated.

At the tender point, at the junction of the second rib with the sternum, there was a well marked pulsation which was heaving, and would raise the fingers when pressed upon it. There was also a greater swelling or expansion of this part of the chest than of the corresponding part of the left side. Both sounds of the heart could be heard here as distinctly as over the heart, but different in character. They were both altered, roughened, without being either a whiffing or bellows sound, or a rasp. No aneurismal thrill could be heard or felt. The pulsation of the carotids of the two sides perfectly similar.

She was cupped, and afterwards blistered for the pain in the left axilla; had also the neutral mixture. She was much relieved of this during the next two or three days, and was enabled to breathe more freely; her pulse fell, and the heat of the skin diminished without her gaining any strength.

Wednesday night, Jan. 31, she was taken with sudden and very great difficulty in breathing, so that she was obliged to sit up in bed, and for some time feared immediate death. Not being sent for by the nurse, I did not see her until the morning visit. She was then somewhat relieved, but evidently suffering greatly, and the perspiration standing in large drops all over her face. The pain and prominence at the top of the sternum had increased very much. The pulsation could be felt, as before, at the second rib, and also as low down as the fifth rib, but much diminished from what it was at the second rib. The sounds over the tumour were not clearly divisible into two, but were of a continuous rumbling nature. Pulse very feeble, not counted. Her prostration prevented a more minute examination into the condition of the lungs. I should say also, that during the night she spat largely of blood and mucus.

The palliative was the only course of treatment that could properly be adopted. The night of February 2d, she became insensible, and lay so until Sunday morning (the 4th), two o'clock, when she died.

*Post-mortem examination twelve hours after death.*

On taking up the sternum, we found the right side of the chest occupied by a large mass or tumour, instead of the lung, which was flat on percussion, and accounted also for the bulging of the chest. The upper part of this was so adherent to the first, second, and third ribs, that portions of them were removed with it. The left lung crepitant throughout. There was an effusion of lymph on the pleura at the point corresponding with the pain in left axilla, about the size of a dollar.

The right lung was covered with lymph, the result of old pleurisy, and was more or less adherent throughout, except at its upper, inner, and posterior part. It was crepitant, though in a moderate degree, in the upper and middle lobes, while in the lower lobe it was consolidated and thinned by compression against the posterior part of the chest, by large recent coagula of blood which had been effused into the pleura anterior to the lung. The heart was somewhat dilated, but not hypertrophied. The *descending* aorta healthy from the arch. The *arch* and the *ascending* portion were dilated and studded with several large plates of bone. Just at the origin of the arteria-innominata was found the orifice of an aneurism, which was about an inch and a half in diameter. It was this which filled up all of the upper portion of the anterior part of the right chest, extending from the sternum to the axilla, and from the first to the fifth rib, and pressing the lung posteriorly. At the lower portion of the sac was found the orifice where it had given away, and thence the effusion of blood which had compressed the lower lobe of the right lung. This probably occurred the Wednesday night she was taken worse, and therefore three days before her death. The aneurismal sac was filled with coagula, from the most of which the red particles had been removed, and which could be separated into layers.

The *parietes* of the sac consisted of a dilatation of the middle and external coats, and probably of the internal coat also, of the aorta. Its inner surface was rough, and studded with bony particles precisely like the inner surface of the ascending and arch of the aorta.

The left ventricle was found empty; the right ventricle had coagula of blood in it, which extended also into the pulmonary artery.

